# Young women’s leisure time physical activity determinants: a mixed methods approach

**DOI:** 10.3389/fpsyg.2024.1281681

**Published:** 2024-02-16

**Authors:** Uxue Fernandez-Lasa, Olaia Eizagirre-Sagastibeltza, Ruth Cayero, Estibaliz Romaratezabala, Judit Martínez-Abajo, Oidui Usabiaga

**Affiliations:** ^1^Society, Sport and Physical Activity Research Group (GIKAFIT), Department of Physical Education and Sports, Faculty of Education and Sport, University of the Basque Country (UPV/EHU), Vitoria-Gasteiz, Spain; ^2^Department of Physical Education and Sports, Faculty of Education and Sport, University of the Basque Country (UPV/EHU), Vitoria-Gasteiz, Spain; ^3^Department of Musical, Plastic and Physical Expression Didactics, Faculty of Education and Sport, University of the Basque Country (UPV/EHU), Vitoria-Gasteiz, Spain

**Keywords:** leisure, feminism, exercise, motivations, barriers, socioecological approach

## Abstract

**Introduction:**

The aim of the study was to analyze the habits, motives and barriers related to Leisure Time Physical Activity (LTPA) among young women of Gipuzkoa, from a mixed approach.

**Methods:**

A total of 526 women aged 18–29 (24.60 ± 3.30 years) responded to the Gipuzkoa Women’s Physical Activity Questionnaire (GWPAQ), seven of which were later interviewed.

**Results:**

The main motives for LTPA were intrapersonal — related to health and enjoyment — and to interpersonal networks. However, the main barriers facing LTPA were mostly intrapersonal, such as lack of time, tiredness, and laziness. Contextual factors such as the availability of safe spaces, previous negative experiences, or negative self-perception of motor competence also emerged as conditioning factors in young women’s LTPA habits.

**Discussion:**

This study may help to promote policies aimed at incentivizing LTPA for young women based on their needs and interests, by addressing the diversity of factors.

## 1 Introduction

Physical inactivity is one of the greatest public health concerns of the present century (Blair, 2009). So much so, that the lack of physical activity (PA) and sedentary behaviors have become a key factor in the appearance of chronic noncommunicable diseases (Booth et al., 2017). Based on this global trend (Guthold et al., 2018), increasing the level of PA practice has become a priority intervention strategy to limit damage to health and well-being (Bull et al., 2020). Thus, the World Health Organization (WHO) designed the Global Action Plan on Physical Activity 2018–2030 (World Health Organization, 2019) and established guidelines to increase PA and decrease sedentary behaviors (World Health Organization, 2020).

Benefits of PA practice include its potential for the prevention and control of chronic diseases (Anderson and Durstine, 2019; Bull et al., 2020), as well as multiple physical, psychological, or social ones (Eime et al., 2013). However, despite having ascertained the benefits of PA, the world population faces numerous barriers that limit engagement in PA (Martins et al., 2015; Spiteri et al., 2019). In this regard, García-Hermoso et al. (2023) have concluded that only 17% of adults meet the World Health Organization (2020) guidelines for aerobic and muscle-strengthening activities and particularly stress the low prevalence among women.

According to the European Health Survey in Spain (EESE) (Instituto Nacional de Estadística, 2021), the percentage of men aged 15 and above who engage in regular physical exercise in their free time is higher than that of women — 31.4% versus 21.9%. In addition, 40.6% of women stated that their free time is almost entirely spent in a sedentary manner (Instituto Nacional de Estadística, 2021). There are several factors linked to this situation, as society does not associate women with sport or characteristics related to hegemonic masculinity such as strength, aggressiveness, or competitiveness (Palaščáková and Palaščáková, 2020). Those gender stereotypes are, therefore, another conditioning factor that contributes to the lesser practice and abandonment of PA by women (Chalabaev et al., 2013). Likewise, most women take on caregiving tasks and put other people’s needs before their own, resulting in little energy or time for PA (Samdhal, 2013). Consequently, women enjoy less free time and are more engaged in caregiving tasks, which makes their time management more complex (Brown and Bowmer, 2019).

Regarding the leisure time physical activity (LTPA) habits of women, a study conducted in Gipuzkoa (Basque Country, Spain) found that 32% of women were inactive and 68% were active (Eizagirre-Sagastibeltza et al., 2022), according to WHO guidelines (2020). Among the main reasons for practice, those related to health and physical fitness and entertainment stood out (Eizagirre-Sagastibeltza et al., 2022). In contrast, the main barriers were lack of time, laziness, tiredness due to work, and family responsibilities (Eizagirre-Sagastibeltza et al., 2022). The importance of the collaboration of family, partners and friends in time management was key and found to favor the practice of LTPA, as in other studies (Martín et al., 2022).

According to several studies, there are some life stage-specific factors among young women that have a particular influence on physical inactivity: school completion and emancipation (van Houten et al., 2017), cultural definitions of femininity (Krane et al., 2004), and family relationships such as moving in with a partner or motherhood (Bell and Lee, 2005). Young women live in a time of transition towards adulthood and are often expected to change aspects related to their place of residence, employment status, personal relationships, and motherhood (Bell and Lee, 2005). In contrast, another study indicated that the structuring of their life influences all the activities they perform, well beyond their studies and employment (O'Dougherty et al., 2012). In this context, the main reasons for practicing LTPA among young women reported in the study by Rodriguez-Romo et al. (2018) were related to health and socialization. However, the main barriers to LTPA practice according to other authors are lack of time, employment, and parenting, as it has also been found among older women (Martín et al., 2022). Despite this, other studies associate body image aspects and body aesthetics as factors in young women’s engagement (or not) in LTPA (Flintoff and Scraton, 2001).

This work is based on feminist theories of leisure—since poststructuralist feminism allows us to understand the ways in which women identify and face the difficulties they encounter in their leisure time—and has placed women at the center of the study and considered their diverse realities (Giblin, 2016). When promoting LTPA, it is important to take this diversity into account, regarding women as a heterogeneous group with different realities (Henderson and Gibson, 2013). This will enable the identification of the barriers and difficulties faced by women in these contexts and to tackle them through resilience and empowerment (Henderson, 2013).

Many are the factors that determine the LTPA habits of each person, so understanding them can help boost LTPA (Bauman et al., 2012). Socio-ecological approaches can be very useful to analyze the physical (in) activity of a population (Sallis et al., 2008). Indeed, they help to understand the complex relationship between an individual and his or her physical and sociocultural environment, distinguishing intrapersonal (e.g., health status, motivation), interpersonal (e.g., support from family or friends) and contextual factors (e.g., climate, infrastructures) (Sallis et al., 2006). Socio-ecological approaches also contemplate the different aspects which can impact LTPA behaviors and support the fact that considering the many agents for LTPA promotion can help increase the effectiveness of programs (Sallis, 2018). Therefore, the aim of this study was to analyze the habits, motives, and barriers to LTPA practice among young women in Gipuzkoa, from a mixed approach.

## 2 Methods

A mixed methodology was used, integrating descriptive quantitative methodology and interpretative qualitative methodology in order to agglutinate and interpret data from both research perspectives and delve into a large number of variables (Creswell and Plano, 2018). Thus, two techniques, a questionnaire and focus groups, were employed simultaneously during data collection.

### 2.1 Participants and instruments

The inclusion criteria for participation in the study were being female, being aged between 18 and 29 and residing in the region of Gipuzkoa (Basque Country, Spain). Participation was voluntary and included a signed informed consent form. The research was approved by the Ethics Committee for Research on Human Subjects (CEISH) of the UPV/EHU (M10_2020_296).

In the quantitative section, 526 women (76.43% active and 23.57% inactive) (95% confidence level and 4.24% margin of error) aged 18–29 (24.60 ± 3.30) responded anonymously to the previously validated Gipuzkoa Women’s Physical Activity Questionnaire (GWPAQ) online (Eizagirre-Sagastibeltza et al., 2022).

In the qualitative section, 7 women from different municipalities of Gipuzkoa (3 active and 4 inactive) and aged between 18 and 29 were interviewed using the focus group technique. The discussion groups lasted 60–90 min and were audio-recorded.

### 2.2 Data analysis

The descriptive results are presented as frequencies and percentages. Pearson’s Chi-squared test (χ2) was performed with a statistical significance of *p* < 0.05 to analyze differences between the motives and barriers to LTPA practice and among the PA level-based groups of participants. Effect size (ES) was calculated by attending to Cramér’s *V* when measuring the strength of association between nominal variables, where ES of 0.1, of 0.3, and of 0.5 were considered low, medium, and large, respectively. Cohen’s D was calculated when analyzing the standardized mean difference, where ES of 0.2, of 0.5, and of 0.8 were considered low, medium, and large, respectively. Statistical analysis was performed with the Statistical Package for Social Sciences (SPSS Inc., version 28.0, Inc. Chicago, Illinois, USA).

In the qualitative section, once the transcription of the focus groups was completed, a content analysis was carried out (Gibbs, 2013). The data were read several times to code them inductively, though the three main dimensions and the categories of the socio-ecological model based on the Sallis et al. (2006) model were respected. Specifically, axial coding was performed, in which the categories are related, culminating in the construction of a categorical system of an inductive-deductive nature (Gibbs, 2013). The emergent themes were LTPA habits, LTPA motives, and barriers to LTPA. Within each category, subcategories corresponding to intrapersonal, interpersonal, and contextual dimensions were organized. The entire analysis process was carried out with the computer software Nvivo 12 (QSR International). During the analysis, the anonymity of the participants was preserved.

## 3 Results

### 3.1 LTPA – related habits and sedentary behavior

Table 1 shows the descriptive results for all the participating women regarding minutes of LTPA according to practice intensity. Of the 526 women who responded to the questionnaire, 76.4% (402 women) stated they were active, while 23.6% (124 women) fell into the inactive category according to the WHO guidelines (2020). Most of the women participating in the study were active (76.4%), with over 6 h (390.2 ± 204.7 min) of LTPA at a mostly low-medium intensity (151.1 ± 181.4 and 142.3 ± 130.9 min respectively). Significant differences were observed for all LTPA intensity levels analyzed (low, medium, high; *p* < 0.01, TE = −0.68 to −1.68, moderate to large), as well as for total LTPA per week (*p* < 0.01, TE = −0.85, large).

**Table 1:** Results for all women relative to LTPA minutes as a function of practice intensity level.

	All	Active	Inactive	*p*	Cohen’s *D*
LTPA intensity level (min)					
Low intensity	124.7 ± 165.2	151.1 ± 181.4	43.2 ± 34.1	**	−1.68
Moderate intensity	115.6 ± 124.4	142.3 ± 130.9	33.5 ± 38.2	**	−0.68
Vigorous intensity	77.2 ± 108.3	98.5 ± 115.9	11.9 ± 29.5	**	−0.94
Total weekly LTPA	316.3 ± 221.1	390.2 ± 204.7	88.5 ± 39.7	**	−0.85

LTPA, Leisure Time Physical Activity; p, *T*-test results for independent samples.^**^p < 0.01 significant differences as a function of minutes of LTPA practice between active and inactive women.

In regard to the number of hours that participants spent sitting per day during work and/or study (Table 2), 80.8% of them responded that they sat for more than 3 h per day, and almost half of them (42.4%) reported sitting for over 6 h per day (40.3% in the case of active women and 49.2% in the case of inactive women), with significant differences observed in the time spent sitting between the two groups (*p* < 0.001). Regarding sedentary behaviors during leisure time, the most common response was spending 1–2 h engaging in sedentary activities, for both groups (43.3% active and 32.3% inactive). Significant differences were found in the time spent doing sedentary activities during leisure time between active and inactive women (*p* < 0.001). These differences were observed up to the range of 3–4 h of sedentary behavior (*p* < 0.001 and *p* < 0.005), since beyond this range there were no significant differences between active and inactive women (*p* > 0.05). In addition, inactive women showed a greater tendency to remain sedentary for more hours than active women.

**Table 2:** Results for all participants in terms of hours spent sitting daily, whether working, studying or sedentary during leisure time.

	All	Active	Inactive	*p*	*Cramer’s V*
Sitting during work/study				**	
Sitting <1 h	13 (2.5%)	8 (2.0%)	5 (4.0%)		0.176
Sitting 1–2 h	38 (7.2%)	30 (7.5%)	8 (6.5%)	**	0.017
Sitting 2–3 h	50 (9.5%)	44 (10.9%)	6 (4.8%)	**	0.088
Sitting 3–4 h	54 (10.3%)	42(10.4%)	12 (9.7%)	**	0.011
Sitting 4–5 h	71 (13.5%)	66 (16.4%)	5 (4.0%)	**	0.154
Sitting 5–6 h	77 (14.6%)	50 (12.4%)	27 (21.8%)	**	0.112
Sitting >6 h	223 (42.4%)	162 (40.3%)	61 (49.2%)	**	0.076
Sedentary behavior during leisure				**	
Sedentary behavior for <1 h	101 (19.2%)	87 (21.6%)	14 (11.3%)	**	0.112
Sedentary behavior for 1–2 h	214 (40.7%)	174 (43.3%)	40 (32.3%)	**	0.095
Sedentary behavior for 2–3 h	120 (22.8%)	93 (23.1%)	27 (21.8%)	**	0.014
Sedentary behavior for 3–4 h	46 (8.7%)	31 (7.7%)	15 (12.1%)	*	0.066
Sedentary behavior for 4–5 h	23 (4.4%)	9 (2.2%)	14 (11.3%)		0.188
Sedentary behavior for 5–6 h	10 (1.9%)	3 (0.7%)	7 (5.6%)		0.152
Sedentary behavior for >6 h	12 (2.3%)	5 (1.2%)	7 (5.6%)		0.125

p, results of Pearson’s Chi-square test, **p < 0.01 and *p < 0.05 significant differences in terms of hours sitting per day both working and sedentary in leisure time between active and inactive women.

While regarding the WHO guidelines, some participants of the focus groups stated that they were physically active and highlighted that they practiced a lot of LTPA, while others stated that they were totally inactive despite having been active in their childhood. The latter emphasized that due to life changes and during the transition from adolescence to adulthood there was a change in LTPA habits, sometimes involving an increase in sedentary time and in other cases involving the total abandonment of LTPA.

### 3.2 Motives for LTPA

Table 3 shows that motives linked to intrapersonal factors (83.7%) were more important than interpersonal factors (14.9%) or contextual factors (1.3%). The main intrapersonal motives for LTPA reported by women were being fit (61.4%), enjoying exercise (54.2%) and the sense of personal accomplishment obtained through exercise (42.5%). Among the interpersonal factors, being in contact with friends and people they enjoy (38.3%), participating in social activities (16.7%) and sharing activities with other women (9.5%) stood out. The most common context-related reason behind LTPA was as a consequence of confinement during the pandemic (26.6%).

**Table 3:** Results of motives for LTPA of active women.

**Factor**	**Motives**	***N***	**%**	**Factor *N* (%)**
Intrapersonal	Be fit	247	61.4	1,522 (83.7%)
	Exercise is good entertainment for me	218	54.2	
	Physical activity gives me a sense of personal accomplishment	171	42.5	
	Avoid or manage health conditions	162	40.3	
	Improve athletic performance	161	37.5	
	Improve my body’s appearance	151	34.5	
	Improve mood	139	34.3	
	Lose or maintain weight	138	33.5	
	Improve my self-esteem	135	38.3	
Interpersonal	Physical activity lets me have contact with friends and people I enjoy	154	38.3	272 (14.9%)
	Participate in social activities	67	16.7	
	Sharing activities with other women	38	9.5	
	Exercising increases my acceptance by others	9	2.2	
	Play with children/ grandchildren/ nephew/ niece	4	1.0	
Contextual	As a consequence of the confinement during the pandemic	33	26.6	24 (1.3%)

Several intrapersonal motives stand out in the interview analysis. The first had to do with the daily organization of LTPA and the second was related with personal commitment. Some of the subjects had internalized routines; thus, GD2 Bidasoa said: “the habit of walking is very widespread. It also happens to me, I like going to Olarizu, by myself. I put on music, and it’s usually a moment of disconnection. In this case I rather go alone, it’s a moment for myself.” Others acknowledged that they needed some kind of obligation or commitment, which they acquired when they signed up for specific activities or sports centers. In this sense, GD1 Oiartzun said: “if there’s a commitment, if I sign up for something, then it’s easier, because I have to go.”

Among the interpersonal motives, the support received from the people around them and the importance of belonging to a group stood out. Interpersonal networks appeared as an important reason for practice, as the women felt more motivated if they had support, and it was easier for them to take the step towards LTPA and to access spaces where they did not feel so comfortable. “What happens to me is that I need a commitment. Come on, I’m going to the mountain! And I cannot, I’d never to do it alone” (GD1 Oiartzun). They recognized that one of the best cures for laziness is to meet up with someone for some LTPA. “Sometimes, just by looking at the weather forecast one may not want to go. But then, if you have arranged to meet with friends, you already have that pressure, and you are encouraged” (GD2 Bidasoa). It can be interesting to create networks, to promote sports associations. For example, GD2 Urola said: “I have not known the “Emakumea Pilotari” [Women Pelotari] initiative, but in an organized group that encourages the participation of women one feels more motivated. Bringing together the local people, the local women, and doing activities together. If something motivates me it’s getting together with women of different ages. If sports were in encouraged in these types of groups, I think I’d join” (GD2 Urola).

Regarding contextual motives, participants underlined several socio-cultural motivations that are culturally accepted and closely related to certain practices of LTPA: “If we go for a hike, let us not fool ourselves, it’s to have a nice lunch afterwards! hahaha...” (GD1 Oria). Moreover, during the post-pandemic stage, LTPA in nature increased considerably and has become a social trend: “it’s become very fashionable, it seems like we are all mountaineers now!” (GD2 Errobi). In this sense, they explained that living close to nature is an advantage. “I’ve realized that I value being in nature, that I’ve somehow achieved a connection with it, I need that contact” (GD2 Bidasoa). However, at the same time, these young women also seek to empower themselves in institutionalized spaces. Thus, they become aware of the need to occupy various sports spaces and understand that awareness is the first step to achieve their goal. They also stressed that this awareness is even greater when it is built collectively. This was expressed by two interviewees:

*We need to regain confidence, to feel good while doing sports, without anyone conditioning or judging us. I think it’s necessary to achieve this, at least for me, it’s necessary to build comfortable and safe spaces for women (GD2 Añarbe).*

*I think we should create women-only spaces to empower ourselves. Once that’s achieved, we’ll see what the next steps should be (GD2 Urola).*

### 3.3 Barriers to LTPA

Barriers linked to intrapersonal factors (85.6%) were more important than the rest of the factors analyzed. Likewise, contextual barriers (7.7%) had a greater weight than interpersonal ones (6.7%). The main intrapersonal barriers were lack of time (66.9%), fatigue due to work or studies (54.8%) and laziness (49.2%). Among the interpersonal barriers, having no one to go with (25.8%) and not feeling comfortable with the people who exercise with them (4.8%) stood out. Lastly, among the contextual barriers, being discouraged by the weather (26.6%), having no adequate facilities (5.7%) and lack of transportation (2.4%) (Table 4) can be highlighted.

**Table 4:** Results of barriers to LTPA for inactive women.

Factor	Barriers	*N*	%	Factor *N* (%)
Intrapersonal	Lack of time	83	66.9	489 (85.6%)
	Fatigue due to work or studies	68	54.8	
	Laziness	61	49.2	
	Overwork	55	44.4	
	Prefer to do other things	39	31.5	
	I am embarrassed to exercise	38	30.7	
	Lack of confidence	36	29.0	
	I do not like doing exercise	26	21.0	
	Ill health, injury, or disability	18	14.5	
	Feeling that my physical appearance is worse than that of others	17	13.7	
	Sense of insecurity (darkness, unknown areas)	16	12.9	
	I feel too fat/overweight	13	10.5	
	Lack of money	12	9.7	
	I think I look ridiculous in exercise clothes	7	5.7	
Interpersonal	I have nobody to go with	32	25.8	38 (6.7%)
	I am not comfortable with people exercising with me	6	4.8	
Contextual	The weather puts me off	33	26.6	44 (7.7%)
	Lack of adequate facilities in my area	7	5.7	
	Lack of transport	3	2.4	
	Lack of suitable monitors/trainers	1	0.8	

From the interviews, it was found that young women encounter intrapersonal barriers that condition their LTPA practice, such as low physical self-concept, previous experiences, feelings of loneliness, negative feelings, and lack of confidence. Physical self-concept may be one of the factors conditioning LTPA. GD1 Oria said: “during my life I have not been good at sports, I have not been a good athlete. In the end it becomes a vicious circle, does not it? Because you do not see yourself as fit, you do not do much, and then you’ll never be fit!.” In this sense, GD1 Urumea added that “you feel very observed and at risk of being criticized.” The interviewees showed the desire for current Physical Education to work on non-competitive practices and observed a change with regard to what they had experienced in the past. “We used to do gymnastics and competitive sports with the objective of improving marks. In the end it’s a bit like in math: if you are good, well, great! In Physical Education it was the same, besides, being physically skilled is a quality that society values” (GD1 Oria).

Some girls felt loneliness when practicing LTPA, which influenced their motivation. This was expressed by GD2 Deba: “Personally, it’s very difficult for me to go alone, I need another person, or to go in a group.” Along the same lines, some felt embarrassed or afraid to get together with other people, due to a low perception of their motor competence. In reference to this, GD2 Deba stated the following: “you think that maybe you will not be able to go to the mountains with people who have a different physical condition and that is why you do not dare.” They do not want to bother or feel out of place. “We’re afraid of feeling like a nuisance, so that’s why we decided not to go” (GD2 Bidasoa). “At paddle tennis matches, people are serious, and you hear comments like, “what a boring match!” So, I do not go so as not to be a nuisance” (GD2 Bidasoa). They recognized that they must overcome personal limitations such as lack of confidence. “I’m very aware of the little relationship I have with physical activity, and it has been difficult for me to feel good doing sports, because of my distrust and so on” (GD1 Urumea).

Furthermore, they reflected on the interpersonal barriers they encounter when practicing LTPA: low expectations from the environment, sexist attitudes from peers, different levels of physical condition and lack of adherence. Thus, they acknowledged having had experiences that did not further encourage them, comments from important people in their lives that showed low expectations towards them.

*Since we’re little, we’re used to hearing certain messages because we’re women, leading us to somehow learn what our place in sport is. So, we maybe take up peripheral sports, ones that are not very important. If we had different demands, things would be different (GD2 Urola).*

The situation is such that they felt that they were perceived as less physically skilled. “There was a coach who on the first day of training came to me and told me how I had to do things, without even asking me if I knew how to do it or what my goal was. I did not like it” (GD2 Añarbe). Likewise, they heard discouraging and demotivating comments, as GD1 Urumea stated: “then, sometimes you hear “you are not good” and that does not help.” Additionally, the sexist attitudes of men of the same age undermine the motivation of young women. This was stated by GD2 Urola:

*In the 7-a-side soccer championship we can participate, and we girls sign up as a group, we have the option to compete against boys. But their response when it’s their turn to play against us is “Damn it!” They play reluctantly against the girls. This shows that, although the options are the same a priori, the role of each gender is very different (GD2 Urola).*

The support of other people was important to the interviewees, and they stressed that they valued it more when it came from others who had a similar level of fitness to their own. In this sense, they showed their concern when deciding whom to go with “When I go with Maddi she gets cold, and I feel bad about it. I need someone who has a more similar level to mine to be more at ease” (GD1 Urumea). With all these drawbacks, they recognized that it was difficult for them to maintain the routine. This lack of stickability is reflected in the words of GD2 Bidasoa, who compared the girls’ adherence to the LTPA with that of the boys: “most of the groups of girls who signed up to try 7-a-side soccer stayed for 3 years and then quit. On the other hand, the boys continue” (GD2 Bidasoa).

As for contextual barriers, unfriendly spaces, the need for adequate pedagogy, the disadvantages of living in small towns or the absence of sports offerings with a gender perspective become relevant. The interviewees stated that it was difficult for them to face a space they considered unfriendly and that their absence did not have much influence on the environment.

*Many of the girls in our friend group have never signed up [for sports practice] because they don't want to, they’ve never played soccer and don't want to do it against boys and in front of the whole town. They don't feel comfortable, and they know what's in that championship. They’re not encouraged. In the last few years my team has not been out, and I’ve had to play in another one (GD2 Deba).*

They emphasized the need for pedagogy. “We must teach people and women who are not used to doing sports that when they start, by taking their time, they can adapt and become good at the sport” (GD2 Bidasoa). In this sense, GD1 Urumea made the following comparison to emphasize that a multilevel offer could facilitate sports practice: “in my opinion, it should be as with language levels: A1, A2, B1, B2... and everyone can choose what best works for them.”

In reference to the sports offer, although they acknowledged that it was broad, they missed a greater gender perspective.

*Today we have options, but that doesn’t mean that we’re given the same importance, right? Here in the soccer club, you can clearly see the conditions that women and men soccer players have had over the years. They’re not the same, that’s for sure (GD2 Añarbe).*

## 4 Discussion

### 4.1 LTPA-related habits and sedentary behavior

The aim of this study was to analyze the habits, motives, and barriers to LTPA practice among young women from Gipuzkoa, from a mixed approach. Regarding habits, approximately two thirds of the women participating in the study were active, matching the results of other scientific studies (World Health Organization, 2020; García-Hermoso et al., 2023). As for the intensity of the activity performed, most was of low-medium intensity, also in agreement with the findings of Moreno-Llamas et al. (2021).

Concerning daily time spent sitting at work and/or studying, the results of the present study agree with those found in other studies (Moreno-Llamas et al., 2021), and highlight the many hours spent sitting by inactive women, with periods of over 3 h. Attitudes towards sedentary behavior are broad and depend on individual aspects and previous experiences (Landais et al., 2022). In the words of Chau et al. (2013), sitting for many hours per day and these sedentary behaviors can be detrimental to health, so understanding the factors that determine them is important for the development of public health strategies aimed at reducing sedentarism among the population (de Victo et al., 2023).

### 4.2 Motives for LTPA

Intrapersonal motives for LTPA were the most frequent among active young women. Particularly, they were related to health status, sports performance, personal development, or physical appearance, which matches the results of other studies (Caglar et al., 2009; Hoare et al., 2017; Sukys et al., 2019). Concern for aesthetics and body image is the result of the social pressure to which women are exposed (Moreno-Murcia et al., 2016), stemming from imposed female beauty standards (Bhatnagar et al., 2021). In the interviews, women pointed to individual organization and personal commitment towards PA as motivational elements. LTPA may be a challenge that women can overcome with practice and effort, which gives them a feeling of satisfaction and self-improvement (Hulteen et al., 2017), and a motive to continue with said practice.

In terms of interpersonal reasons, one of the main factors was socialization. The commitment acquired with a group of people during LTPA enhances the group feeling and individual satisfaction of each of the group members. This can drive an empowerment process to challenge and transform LTPA for more women through this collective awareness, as proposed by Fernandez-Lasa et al. (2020). Moreover, as Taylor (2014) concluded, many young women seem to feel more comfortable and motivated when they group exclusively with other women for LTPA, which reinforces the idea of generating women-only intervention programs. The latter fact is evident among the interviewees, who stressed the need for a supportive environment where they could make a commitment to the group. Physical activity helps in the psychosocial development demanded by women, offers opportunities for interaction with other women, and creates a feeling of belonging and a sense of community (Moreno-Murcia et al., 2016).

Among the contextual reasons, it appears that pandemic-related restrictions encouraged the women in this study to practice LTPA in nature. When they were not able to enjoy nature due to confinement was when they valued it most. Interviews suggest that these activities have become “fashionable” and highlight the need to be in contact with nature. According to Calogiuri and Elliott (2017), nature based LTPA is motivated by extrinsic factors related to the tranquility brought by nature itself, and which are different from the intrinsic motivations found in other contexts such as the gym or sports that are not performed in nature. Interviewees also expressed the need to promote spatial empowerment, so women feel comfortable and safe. Thus, they stressed the need to create spaces and activities exclusively for women. Along the same lines, Saavedra (2009) considered sports as a means of empowerment where opportunities are created for women to participate freely.

### 4.3 Barriers to LTPA

Inactive young women aged 18–29 mostly highlighted intrapersonal barriers that influenced their lower engagement in LTPA. This is consistent with other studies where similar barriers—lack of time, tiredness, laziness, overwork, other leisure preferences, embarrassment, or lack of confidence—were observed (Hoare et al., 2017; Ferreira-Silva et al., 2022). In addition to these confirmed barriers, interviewees underlined the importance of previous negative experiences in physical education and sport’s class, in the terms mentioned by Cardinal et al. (2013), and the fear of going out alone, as also found by Sreetheran and Van Den Bosch (2014).

One of the main interpersonal barriers expressed by inactive young women was the difficulty to find other people with whom to practice LTPA. In this sense, Abbasi (2014) concluded that social isolation was a socio-cultural barrier that prevented women from reaching the recommended levels of PA. Other barriers were also relevant for the interviewees, such as the awkwardness (fear of judgment) generated by practicing LTPA in public spaces or the little help they perceived from men of the same age, as also highlighted by other authors (Deliens et al., 2015; Seal et al., 2022).

Bad weather was one of the contextual factors that most discouraged young women from LTPA, as is the case with adults in general (Humpel et al., 2002; Tucker and Gilliland, 2007). Additionally, they emphasized the relevance of unfriendly spaces in which they did not feel comfortable, and the need for safe spaces. In this regard, Laatikainen et al. (2017) concluded that space is key for the development of human behavior, so choosing safe, accessible, and, above all, attractive spaces for different age groups can be crucial for LTPA practice (Barnett et al., 2017).

## 5 Conclusion

The use of a mixed approach combining quantitative and qualitative methods may be appropriate to analyze habits, motives, and barriers to LTPA among young women from Gipuzkoa, thus providing richer and more holistic information about their perceptions and experiences.

A quarter of young women from Gipuzkoa are physically inactive during their leisure time and over half of the participants report sedentary behaviors at work and school, which may have a negative impact on their health.

The main motives for practicing LTPA were intrapersonal and related to health maintenance and enjoyment, as well as to social networks of the interpersonal dimension. However, the main contextual motive was linked to the COVID19 pandemic. Other aspects emerged among the qualitative reasons, such as personal and group commitment, peer pressure associated with body image or beauty standards, and the need to carry out activities in nature.

The main barriers to LTPA were intrapersonal, with lack of time, fatigue and laziness being the most reported. Important contextual factors were bad weather, the need to promote safe spaces, the need to adapt the offer of PA activities and sports to the circumstances and interests of the youngest girls, and lacking companions to practice LTPA with. Further, the influence of previous negative experiences in PE and the negative perception of motor competence on future LTPA habits of young women—due to embarrassment and lack of confidence rooted in earlier stages of their lives—should also be highlighted.

The main limitation of this study lies in the difficulty for the recruitment of women for the focus groups, leading to a relatively small number of interviews. Therefore, as a future line of research, it would be interesting to interview women at different life stages to analyze the diversity of their characteristics and study how they influence women’s engagement in LTPA. Moreover, it would be interesting to analyze how the area of residence influences the habits, motives, and barriers to LTPA, since Gipuzkoa is a region with over 50% of semi-urban and rural population.

## Data availability statement

The raw data supporting the conclusions of this article will be made available by the authors, without undue reservation.

## Ethics statement

The studies involving humans were approved by the Ethics Committee for Research on Human Subjects (CEISH) of the UPV/EHU (M10_2020_296). The studies were conducted in accordance with the local legislation and institutional requirements. The participants provided their written informed consent to participate in this study.

## Author contributions

UF-L: Conceptualization, Formal analysis, Investigation, Methodology, Supervision, Writing – original draft, Writing – review & editing, Data curation. OE-S: Conceptualization, Formal analysis, Investigation, Methodology, Supervision, Writing – original draft, Writing – review & editing. RC: Investigation, Methodology, Writing – original draft, Writing – review & editing. ER: Investigation, Software, Writing – original draft, Writing – review & editing. JM-A: Investigation, Methodology, Writing – original draft, Writing – review & editing. OU: Conceptualization, Formal analysis, Investigation, Project administration, Supervision, Writing – original draft, Writing – review & editing.

## References

[ref1] Abbasi I. N. (2014). Socio-cultural barriers to attaining recommended levels of physical activity among females: a review of literature. Quest 66, 448–467. doi: 10.1080/00336297.2014.955118

[ref2] Anderson E. Durstine J. L. (2019). Physical activity, exercise, and chronic diseases: a brief review. Sports Med. Health Sci. 1, 3–10. doi: 10.1016/j.smhs.2019.08.006, PMID: 35782456 PMC9219321

[ref3] Barnett D. W. Barnett A. Nathan A. Van Cauwenberg J. Cerin E. (2017). Built environmental correlates of older adults' total physical activity and walking: a systematic review and meta-analysis. Int. J. Behav. Nutr. Phys. Act. 14:103. doi: 10.1186/s12966-017-0558-z, PMID: 28784183 PMC5547528

[ref4] Bauman A. E. Reis R. S. Sallis J. F. Wells J. C. Loos R. J. Martin B. W. (2012). Correlates of physical activity: why are some people physically active and others not? Lancet 380, 258–271. doi: 10.1016/S0140-6736(12)60735-1, PMID: 22818938

[ref5] Bell S. Lee C. (2005). Emerging adulthood and patterns of physical activity among young Australian women. Int. J. Behav. Med. 12, 227–235. doi: 10.1207/s15327558ijbm1204_3, PMID: 16262541

[ref6] Bhatnagar P. Foster C. Shaw A. (2021). Barriers and facilitators to physical activity in second-generation British Indian women: a qualitative study. PLoS One 16:e0259248. doi: 10.1371/journal.pone.0259248, PMID: 34731201 PMC8565737

[ref7] Blair S. N. (2009). Physical inactivity: the biggest public health problem of the 21st century. Br. J. Sports Med. 43, 1–2. PMID: 19136507

[ref8] Booth F. W. Roberts C. K. Thyfault J. P. Ruegsegger G. N. Toedebusch R. G. (2017). Role of inactivity in chronic diseases: evolutionary insight and pathophysiological mechanisms. Physiol. Rev. 97, 1351–1402. doi: 10.1152/physrev.00019.2016, PMID: 28814614 PMC6347102

[ref9] Brown N. Bowmer Y. (2019). A comparison of perceived barriers and motivators to physical activity in young and middle-aged women. Women Sport Physical Activity J. 27, 52–59. doi: 10.1123/wspaj.2017-0045

[ref10] Bull F. C. Al-Ansari S. S. Biddle S. Borodulin K. Buman M. P. Cardon G.. (2020). World Health Organization 2020 guidelines on physical activity and sedentary behaviour. Br. J. Sports Med. 54, 1451–1462. doi: 10.1136/bjsports-2020-102955, PMID: 33239350 PMC7719906

[ref11] Caglar E. Canlan Y. Demir M. (2009). Recreational exercise reasons of adolescents and young adults. J. Hum. Kinet. 22, 83–89. doi: 10.1186/1471-2458-14-909

[ref12] Calogiuri G. Elliott L. R. (2017). Why do people exercise in natural environments? Norwegian adults’ reasons for nature-, gym-, and sports-based exercise. Int. J. Environ. Res. Public Health 14:377. doi: 10.3390/ijerph14040377, PMID: 28375192 PMC5409578

[ref13] Cardinal B. J. Yan Z. Cardinal M. K. (2013). Negative experiences in physical education and sport: how much do they affect physical activity participation later in life? J. Physic. Educ. Recreation Dance 84, 49–53. doi: 10.1080/07303084.2013.767736

[ref14] Chalabaev A. Sarrazin P. Fontayne P. Boiché J. Clément-Guillotin C. (2013). The influence of sex stereotypes and gender roles on participation and performance in sport and exercise: review and future directions. Psychol. Sport Exerc. 14, 136–144. doi: 10.1016/j.psychsport.2012.10.005

[ref15] Chau J. Y. Grunseit A. C. Chey T. Stamatakis E. Brown W. J. Matthews C. E.. (2013). Daily sitting time and all-cause mortality: a meta-analysis. PloS One 8:e80000. doi: 10.1371/journal.pone.0080000, PMID: 24236168 PMC3827429

[ref16] Creswell J. W. Plano V. L. (2018). Designing and conducting mixed methods research. Thousand Oaks, California: Sage.

[ref17] de Victo E. R. Fisberg M. Solé D. Kovalskys I. Gómez G. Rigotti A.. (2023). Joint association between sedentary time and moderate-to-vigorous physical activity with obesity risk in adults from Latin America. Int. J. Environ. Res. Public Health 20:5562. doi: 10.3390/ijerph20085562, PMID: 37107844 PMC10138536

[ref18] Deliens T. Deforche B. De Bourdeaudhuij I. Clarys P. (2015). Determinants of physical activity and sedentary behaviour in university students: a qualitative study using focus group discussions. BMC Public Health 15, 201–209. doi: 10.1186/s12889-015-1553-4, PMID: 25881120 PMC4349731

[ref19] Eime R. M. Young J. A. Harvey J. T. Charity M. J. Payne W. R. (2013). A systematic review of the psychological and social benefits of participation in sport for children and adolescents: informing development of a conceptual model of health through sport. Int. J. Behav. Nutr. Phys. Act. 10, 98–21. doi: 10.1186/1479-5868-10-98 23945179 PMC3751802

[ref20] Eizagirre-Sagastibeltza O. Fernandez-Lasa U. Yanci J. Romaratezabala E. Cayero R. Iturrioz I.. (2022). Design and validation of a questionnaire to assess the leisure time physical activity of adult women in Gipuzkoa. Int. J. Environ. Res. Public Health 19:5736. doi: 10.3390/ijerph19095736, PMID: 35565129 PMC9103327

[ref21] Fernandez-Lasa U. Usabiaga O. Soler Prat S. (2020). Juggling on the court: exploring female Basque pelota players’ experiences and empowerment strategies. J. Gend. Stud. 29, 496–507. doi: 10.1080/09589236.2019.1618703

[ref22] Ferreira-Silva R. M. Mendonça C. R. Azevedo V. D. Raoof-Memon A. Noll P. R. E. S. Noll M. (2022). Barriers to high school and university students’ physical activity: a systematic review. PLoS One 17:e0265913. doi: 10.1371/journal.pone.0265913Ls, PMID: 35377905 PMC8979430

[ref23] Flintoff A. Scraton S. (2001). Stepping into active leisure? Young women's perceptions of active lifestyles and their experiences of school physical education. Sport Educ. Soc. 6, 5–21. doi: 10.1080/713696043

[ref24] García-Hermoso A. López-Gil J. F. Ramírez-Vélez R. Alonso-Martínez A. M. Izquierdo M. Ezzatvar Y. (2023). Adherence to aerobic and muscle-strengthening activities guidelines: a systematic review and meta-analysis of 3.3 million participants across 32 countries. Br. J. Sports Med. 57, 225–229. doi: 10.1136/bjsports-2022-106189 36418149

[ref25] Gibbs G. (2013). El análisis de datos cualitativos en investigación cualitativa, vol. 6. Las rozas de Madrid, Madrid, Spain: Ediciones Morata.

[ref26] Giblin H. (2016). “Feminism, Poststructural” in The Wiley Blackwell encyclopedia of gender and sexuality studies. ed. Bridget Somekh C. L. (Thousand Oaks, California: Sage Publications), 318–325.

[ref27] Guthold R. Stevens G. A. Riley L. M. Bull F. C. (2018). Worldwide trends in insufficient physical activity from 2001 to 2016: a pooled analysis of 358 population-based surveys with 1·9 million participants. Lancet Glob. Health 6, e1077–e1086. doi: 10.1016/S2214-109X(18)30357-7, PMID: 30193830

[ref28] Henderson K. A. (2013). “Feminist leisure studies: origins, accomplishments and prospects” in Routledge handbook of leisure studies. ed. Blackshaw T. (Abingdon, Oxfordshire: Routledge), 26–39.

[ref29] Henderson K. A. Gibson H. J. (2013). An integrative review of women, gender, and leisure: increasing complexities. J. Leis. Res. 45, 115–135. doi: 10.18666/jlr-2013-v45-i2-3008

[ref30] Hoare E. Stavreski B. Jennings G. L. Kingwell B. A. (2017). Exploring motivation and barriers to physical activity among active and inactive Australian adults. Sports 5:47. doi: 10.3390/sports5030047, PMID: 29910407 PMC5968958

[ref31] Hulteen R. M. Smith J. J. Morgan P. J. Barnett L. M. Hallal P. C. Colyvas K.. (2017). Global participation in sport and leisure-time physical activities: a systematic review and meta-analysis. Prev. Med. 95, 14–25. doi: 10.1016/j.ypmed.2016.11.02 27939265

[ref32] Humpel N. Owen N. Leslie E. (2002). Environmental factors associated with adults’ participation in physical activity: a review. Am. J. Prev. Med. 22, 188–199. doi: 10.1016/S0749-3797(01)00426-3 11897464

[ref33] Instituto Nacional de Estadística (2021). Available at: *Encuesta Europea de Salud en España*. https://www.ine.es/dyngs/INEbase/es/operacion.htm?c=Estadistica_C&cid=1254736176784&menu=resultados&idp=1254735573175

[ref34] Krane V. Choi P. Y. L. Baird S. M. Aimar C. M. Kauer K. J. (2004). Living the paradox: female athletes negotiate femininity and muscularity. Sex Roles 50, 315–329. doi: 10.1023/B:SERS.0000018888.48437.4f

[ref35] Laatikainen T. E. Broberg A. Kytta M. (2017). The physical environment of positive places: exploring differences between age groups. Prev. Med. 95, S85–S91. doi: 10.1016/j.ypmed.2016.11.015, PMID: 27986540

[ref36] Landais L. L. Jelsma J. G. M. Dotinga I. R. Timermens D. R. M. Verhagen E. Damman O. C. (2022). Office workers' perspectives on physical activity and sedentary behaviour: a qualitative study. BMC Public Health 22:621. doi: 10.1186/s12889-022-13024-z, PMID: 35354447 PMC8966601

[ref37] Martín M. Barriopedro M. I. Espada M. (2022). Influencia de la edad, la maternidad y el empleo en las barreras para la práctica de actividad física y deporte de las mujeres adultas en España. Retos 44, 667–675. doi: 10.47197/retos.v44i0.88076

[ref38] Martins J. Marques A. Sarmento H. Carreiro da Costa F. (2015). Adolescents’ perspectives on the barriers and facilitators of physical activity: a systematic review of qualitative studies. Health Educ. Res. 30, 742–755. doi: 10.1093/her/cyv042, PMID: 26324394

[ref39] Moreno-Llamas A. García-Mayor J. De la Cruz-Sánchez E. (2021). How Europeans move: a moderate-to-vigorous physical activity and sitting time paradox in the European Union. Public Health 203, 1–8. doi: 10.1016/j.puhe.2021.11.016, PMID: 34968833

[ref40] Moreno-Murcia J. A. Marcos-Pardo P. J. Huéscar E. (2016). Motivos de Práctica Físico-Deportiva en Mujeres: Diferencias entre Practicantes y no Practicantes. Revista Psicol Deporte 25, 35–41.

[ref41] O'Dougherty M. Hearst M. O. Arikawa A. Y. Stovitz S. D. Kurzer M. S. Schmitz K. H. (2012). Young women's physical activity from one year to the next: what changes? What stays the same? Transl. Behav. Med. 2, 129–136. doi: 10.1007/s13142-011-0108-1, PMID: 23482709 PMC3589798

[ref42] Palaščáková D. Palaščáková L. (2020). Persistence of gender stereotypes in sports. J. Women Entrepreneurship Educ. 2020, 72–86. doi: 10.28934/jwee20.12.pp72-86

[ref43] Rodriguez-Romo G. Macías-Pla R. Garrido-Muñoz M. Tejero-González C. M. López-Adan E. (2018). Motivos para la práctica de actividad física durante el tiempo libre y su relación con el cumplimento de las recomendaciones. Cuadernos Psicol. Deporte 18, 183–194.

[ref44] Saavedra M. (2009). “Dilemmas and opportunities in gender and sport-in-development” in Sport and international development. eds. Levermore R. Beacom A. (Crinan Street, London: Palgrave Macmillan UK), 124–155.

[ref45] Sallis J. F. (2018). Needs and challenges related to multilevel interventions: physical activity examples. Health Educ. Behav. 45, 661–667. doi: 10.1177/1090198118796458, PMID: 30122086

[ref46] Sallis J. F. Cervero R. B. Ascher W. Henderson K. A. Kraft M. K. Kerr J. (2006). An ecological approach to creating active living communities. Annu. Rev. Public Health 27, 297–322. doi: 10.1146/annurev.publhealth.27.021405.102100 16533119

[ref47] Sallis J. F. Owen N. Fisher E. B. (2008). “Ecological models of health behavior” in Health behavior and health education: Theory, research and practice. eds. Glanz K. Rimer B. K. Viswanath K.. 4th ed (Hoboken, New Jersey: Jossey-Bass), 465–485.

[ref48] Samdhal D. M. (2013). “Women, Gender, and Leisure Constraints” in Leisure, Women, and Gender. eds. Freysinger V. J. Shaw S. M. Henderson K. A. Bialeschki Y. M. D. (Andover, Massachusetts: Venture Publishing), 109–127.

[ref49] Seal E. Nicholson M. McNeil N. Stukas A. O’Halloran P. Randle E. (2022). Fear of judgement and women's physical (in) activity experiences. Int. Rev. Sociol. Sport 57, 381–400. doi: 10.1177/10126902211016631

[ref50] Spiteri K. Broom D. Bekhet A. H. de Caro J. X. Laventure B. Grafton K. (2019). Barriers and motivators of physical activity participation in middle-aged and older adults—a systematic review. J. Aging Phys. Act. 27, 929–944. doi: 10.1123/japa.2018-0343, PMID: 31141447

[ref51] Sreetheran M. Van Den Bosch C. C. K. (2014). A socio-ecological exploration of fear of crime in urban green spaces–a systematic review. Urban For. Urban Green. 13, 1–18. doi: 10.1016/j.ufug.2013.11.006

[ref52] Sukys S. Cesnaitiene V. J. Emeljanovas A. Mieziene B. Valantine I. Ossowski Z. M. (2019). Reasons and barriers for university students’ leisure-time physical activity: moderating effect of health education. Percept. Mot. Skills 126, 1084–1100. doi: 10.1177/0031512519869089, PMID: 31407961

[ref53] Taylor J. A. (2014). The impact of the ‘girls on the Move’Leadership Programme on young female leaders’ self-esteem. Leis. Stud. 33, 62–74. doi: 10.1080/02614367.2012.727459

[ref54] Tucker P. Gilliland J. (2007). The effect of season and weather on physical activity: a systematic review. Public Health 121, 909–922. doi: 10.1016/j.puhe.2007.04.009, PMID: 17920646

[ref55] van Houten J. M. A. Kraaykamp G. Breedveld K. (2017). When do young adults stop practising a sport? An event history analysis on the impact of four major life events. Int. Rev. Sociol. Sport 52, 858–874. doi: 10.1177/1012690215619204

[ref56] World Health Organization (2019). Global action plan on physical activity 2018–2030: More active people for a healthier world. Geneva, Switzerland: World Health Organization.

[ref57] World Health Organization (2020). *WHO guidelines on physical activity and sedentary behaviour*. Geneva, Switzerland. Available at: https://www.who.int/publications/i/item/9789240015128.

